# [Expression of Concern] *Ganoderma lucidum* polysaccharide inhibits prostate cancer cell migration via the protein arginine methyltransferase 6 signaling pathway

**DOI:** 10.3892/mmr.2026.14010

**Published:** 2026-09-07

**Authors:** Xiaohui Zhao, Dayu Zhou, Yunen Liu, Chun Li, Xiaoguang Zhao, Ying Li, Wei Li

Mol Med Rep 17: 147–157, 2018; DOI: 10.3892/mmr.2017.7904

Following the publication of the above paper, it was drawn to the Editor's attention by a concerned reader that, regarding the histological images shown in Fig. 1B on p. 149, the “Ctrl” and “GLPs 5μg/ml” data panels contained an overlapping section of data, suggesting that these data panels were derived from the same original source where different experimental conditions were reported. In addition, a concern was raised regarding the antibody that had been used to probe for p21 in the western blot experiments in Fig. 5 (cat. no. sc-136020, purchased from Santa Cruz Biotechnology, Inc.), since apparently this antibody is specific for a different protein, sometimes referred to as p21-ARC, which is related to the actin cytoskeleton.

The authors have been contacted by the Editorial Office to offer an explanation for these apparent anomalies in the presentation of the data in their paper, and we are awaiting their response. Due to the fact that we have been made aware of potential issues surrounding the scientific integrity of this paper, we are issuing an Expression of Concern to notify readers of this potential problem while the Editorial Office continues to investigate this matter further.

